# Forest Elephant Crisis in the Congo Basin 

**DOI:** 10.1371/journal.pbio.0050111

**Published:** 2007-04-03

**Authors:** Stephen Blake, Samantha Strindberg, Patrick Boudjan, Calixte Makombo, Inogwabini Bila-Isia, Omari Ilambu, Falk Grossmann, Lambert Bene-Bene, Bruno de Semboli, Valentin Mbenzo, Dino S'hwa, Rosine Bayogo, Liz Williamson, Mike Fay, John Hart, Fiona Maisels

**Affiliations:** 1 Africa Program, Wildlife Conservation Society, Bronx, New York, United States of America; 2 Department of Geography, University of Maryland, College Park, Maryland, United States of America; 3 World Wildlife Fund–Cameroon, Yaoundé, Cameroon; 4 World Wildlife Fund–Dzanga-Sangha, Bangui, Central African Republic; 5 Bangassou Forest Project, Canadian Center for International Studies and Cooperation, Bangui, Central African Republic; Imperial College London, United Kingdom

## Abstract

Debate over repealing the ivory trade ban dominates conferences of the Convention on International Trade in Endangered Species of Wild Fauna and Flora (CITES). Resolving this controversy requires accurate estimates of elephant population trends and rates of illegal killing. Most African savannah elephant populations are well known; however, the status of forest elephants, perhaps a distinct species, in the vast Congo Basin is unclear. We assessed population status and incidence of poaching from line-transect and reconnaissance surveys conducted on foot in sites throughout the Congo Basin. Results indicate that the abundance and range of forest elephants are threatened from poaching that is most intense close to roads. The probability of elephant presence increased with distance to roads, whereas that of human signs declined. At all distances from roads, the probability of elephant occurrence was always higher inside, compared to outside, protected areas, whereas that of humans was always lower. Inside protected areas, forest elephant density was correlated with the size of remote forest core, but not with size of protected area. Forest elephants must be prioritised in elephant management planning at the continental scale.

## Author Summary

Forest elephants, perhaps a distinct species of African elephant, occur in the forests of West and Central Africa. Compared to the more familiar savannah elephant of Eastern and Southern Africa, forest elephant biology and their conservation status are poorly known. To provide robust scientific data on the status and distribution of forest elephants to inform and guide conservation efforts, we conducted surveys on foot of forest elephant abundance and of illegal killing of elephants in important conservation sites throughout Central Africa. We covered a combined distance of over 8,000 km on reconnaissance walks, and we systematically surveyed a total area of some 60,000 km^2^ under the auspices of the Monitoring of the Illegal Killing of Elephants (MIKE) programme. Our results indicate that forest elephant numbers and range are severely threatened by hunting for ivory. Elephant abundance increased with increasing distance from the nearest road, and poaching pressure was most concentrated near roads. We found that protected areas have a positive impact on elephant abundance, probably because management interventions reduced poaching rates inside protected areas compared to non-protected forest. Law enforcement to bring the illegal ivory trade under control, and effective management and protection of large and remote national parks will be critical if forest elephants are to be successfully conserved.

## Abstract 3

A systematic survey of 68,000 km^2^ throughout Central Africa reveals that the forest elephant--distinct from the savannah elephant--is severely threatened by poaching, despite a near universal ban of trade in ivory.

## Introduction

Between 1970 and 1989, half of Africa's elephants (Loxodonta africana), perhaps 700,000 individuals, were killed, mostly to supply the international ivory trade [1]. This catastrophic decline prompted the Conference of the Parties (CoP) to the Convention on the International Trade in Endangered Species of Wild Flora and Fauna (CITES) to list African elephants on Appendix I of the convention, banning the international ivory trade. Today, opinions on the management of African elephants, including their international trade status, are polarized among range states, economists, and wildlife managers [2]. Southern African nations and wildlife managers argue that their ability to control poaching and manage elephants should be rewarded through the harvest and sale of their ivory stocks, thereby generating revenue for conservation programmes. A strong lobby headed by Kenya, the Central and West African nations, and conservationists in these regions maintain that re-opening the trade will increase the demand for ivory and stimulate the resumption of uncontrollable illegal killing of elephants throughout the continent. Among economists, conclusions are equivocal on whether resumption of the trade will have a positive or negative impact on elephant populations [3,4].

Central to an informed resolution of this debate is a clear understanding of the size and trends in elephant populations and rates of illegal killing for ivory across Africa. The status of savannah elephant (L. africana africana) populations in Eastern, Western, and Southern Africa are relatively well known, and most appear to be stable or increasing with generally low poaching rates [5], though in Angola, Mozambique, and Zimbabwe, poaching for ivory may be on the increase [6]. The status of forest elephants *(L. africana cyclotis)* in the vast equatorial forest of Africa remains poorly known because methodological problems and severe logistical constraints have inhibited reliable population surveys and estimates of illegal killing [7]. In African savannahs, both elephant populations and illegal killing can be monitored through aerial surveys [8], whereas an elephant massacre can remain undetected in the depths of the forest.

The forest of Central Africa is of critical importance for elephants, comprising over 23% of the total continental elephant range, and the largest contiguous elephant habitat left on the continent [5]. In 1989, following reconnaissance surveys on foot, the forest elephant population of the Congo Basin was estimated at 172,400 individuals, nearly one third of Africa's elephants at that time [9]. Poaching was rampant in some areas, notably the Democratic Republic of Congo [10] (then Zaire), whereas Gabon's elephants were relatively unaffected [11]. Human activity, particularly road infrastructure, was found to be the major factor influencing the distribution of forest elephants [9,12,13]. Since 1989, no further region-wide surveys have been conducted, despite dramatic increases in logging, road infrastructure development, growing human populations, and conflict [14–16], accompanied by considerable development of the protected areas network and conservation funding [17].

Today, forest elephant population estimates are based on guesswork [5], and inventory and monitoring must be improved for five main reasons: (1) forest elephants may still comprise a significant proportion of Africa's total elephant population [5]; (2) forest elephants are distinctive on morphological, ecological, behavioural, and genetic criteria, constituting at least a subspecies and possibly a distinct species of African elephant [18]; (3) Central Africa's forests are the source of much of the world's illicitly traded ivory [19]; (4) the trade status of ivory from Southern African elephants may have a serious impact on poaching levels in Central Africa due to changes in the dynamics of the international legal and illegal ivory trade [2]; and (5) logging and road development in the Congo Basin are increasing dramatically, which is opening up accessibility both to remaining elephant strongholds and to markets.

**Figure 1:**
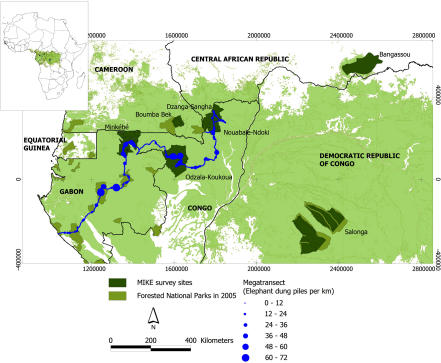
MIKE Survey Sites and the Megatransect Note that the since the Dzanga-Sangha and Nouabalé-Ndoki MIKE sites comprise a contiguous forest block, they were combined into a single unit (Ndoki-Dzanga) for analytical purposes.

During 2003–2005, under the auspices of the Monitoring of the Illegal Killing of Elephants (MIKE) programme and the Projet Espèces Phares of the European Union, we collected data on the distribution, abundance, and illegal killing of forest elephants by means of systematic foot surveys on line transects and reconnaissance walks (see Materials and Methods) at six sites (Figure 1). These MIKE survey sites were centred on protected areas thought to contain nationally important forest elephant populations. We also collected complementary data in 1999 and 2000 on a single, continuous survey of over 2,000 km dubbed the “Megatransect” [20], which ran through some of the most remote forest blocks in Africa (Figure 1). Our goals were to evaluate the conservation status of forest elephants, including population size, distribution, and levels of illegal killing in relation to human activity, isolation from roads, and the impact of protected areas.

## Results

### Forest Elephant Abundance by MIKE Site

Our results indicate that a combination of illegal killing and other human disturbance has had a profound impact on forest elephant abundance and distribution, including inside national parks (NPs). The density of elephants in NPs surveyed varied over two orders of magnitude. In the Salonga NP, a remote United Nations Educational, Scientific, and Cultural Organization (UNESCO) World Heritage site, as few as 1,900 forest elephants remain at a mean density of 0.05 elephant km^−2^. Salonga is the largest forested NP in Africa and the second largest on earth. In Nouabalé-Ndoki and Dzanga-Sangha NPs and their buffer zones (Ndoki-Dzanga MIKE site), 3,900 elephants were estimated within a survey area of 10,375 km^2^ (0.4 elephant km^−2^). Mean estimated forest elephant densities in the three NP sectors at this site were 0.66, 0.65, and 0.56 individuals km^−2^ for Nouabalé-Ndoki NP, Dzanga NP, and Ndoki NP respectively, compared with densities of 0.14 and 0.1 individuals km^−2^ in the peripheral zones of these NPs. In the 2,382-km^2^ Boumba Bek NP in southeast Cameroon, an estimated 318 elephants occurred (0.1 elephant km^−2^). In the Bangassou Forest, one of only two regions in the Central African Republic (CAR) that still contain forest elephants, a formal estimate of elephant abundance was not made, but systematic observations along reconnaissance walks suggest that in the 12,000-km^2^ survey area, fewer than 1,000 forest elephants remain. In only two protected areas, Minkébé NP, northeast Gabon, and Odzala-Koukoua NP, northern Congo, did the mean estimated elephant density exceed 1.0 individual km^−2^. Estimated population size was 22,000 individuals in the 7,592-km^2^ Minkébé NP (2.9 elephants km^−2^) and 14,000 in the 13,545-km^2^ Odzala-Koukoua NP (1.0 elephant km^−2^).

### Elephant Poaching in MIKE Sites

Poached elephant carcasses were found in all MIKE sites, even large, well-established NPs (Table 1). We found 53 confirmed elephant poaching camps and 41 elephant carcasses from 4,477 km of reconnaissance walks; we confirmed 27 carcasses as having been poached. Poached carcass encounter rate was highest in the Minkébé site, at 13.7 carcasses 1,000 km^−1^, followed by Ndoki-Dzanga with 7.1 carcasses 1,000 km^−1^. The tusks had been removed from all poached carcasses, though due to the level of decay, it was not possible to determine whether they had been poached primarily for ivory or for meat.

**Table 1:**
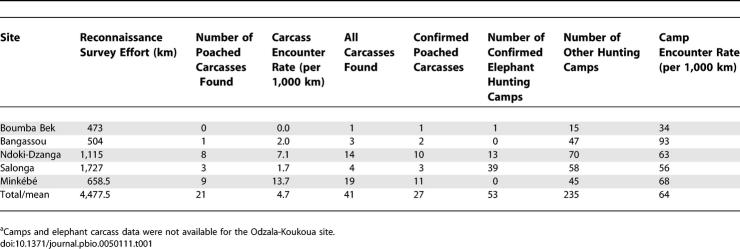
Elephant Poaching Camps and Carcasses Found during Reconnaissance Walks, Line Transects, and Fieldwork-Related MIKE Surveys^a^

### Forest Elephants, Human Activity, and Roads in MIKE Sites

**Figure 2:**
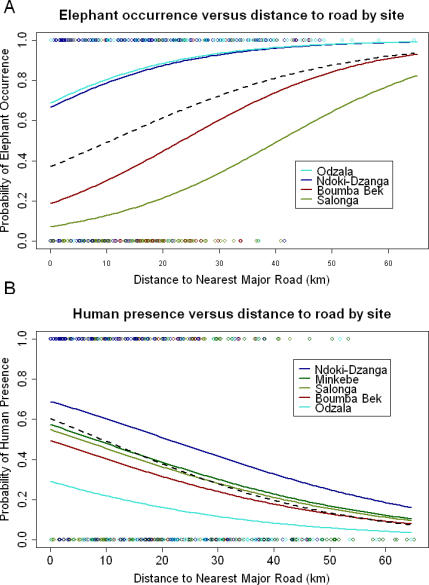
Results of Fitting a Logistic Regression Model to Elephant and Human Presence/Absence Data Pooled across MIKE Survey Sites Distance to road (in kilometres) and site were used as explanatory variables. (A) shows the elephant data, and (B) shows the human data. The observations and regression lines are colour-coded by site and the dashed line shows the regression line without the inclusion of site as a covariate. The covariates distance to road and site are significant for both elephant and human probability of occurrence. The dissimilarity between sites is more pronounced when modelling the probability of elephant occurrence.

**Figure 3:**
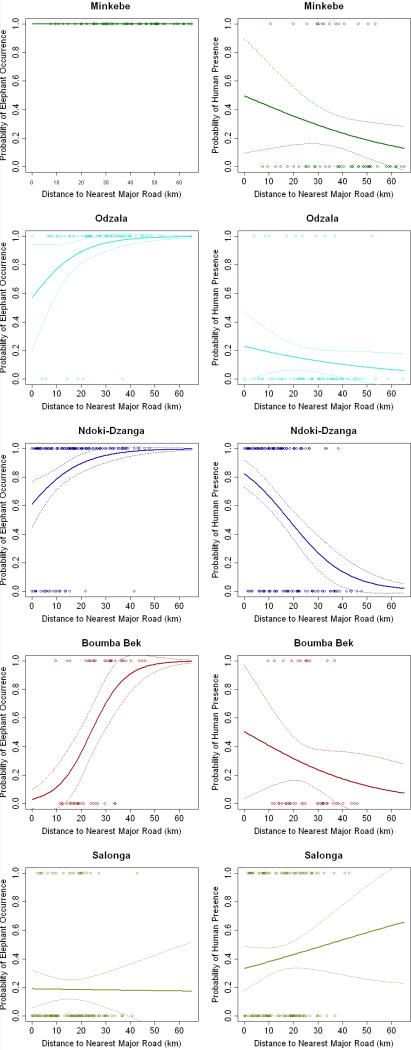
Results of Fitting a Logistic Regression Model to Elephant and Human Presence/Absence Data for Each MIKE Survey Site Separately Distance to road (in kilometres) was used as the explanatory variable (except for probability of elephant occurrence for Minkébé where modelling is not required due to an effective probability of 1). Elephant data are shown to the left, and human data to the right. The observations and regression lines are colour-coded by site, and the 95% confidence interval is indicated by the dotted lines. The probability of elephant occurrence is significantly related to distance to road for all sites except Minkébé and Salonga. Due to the imprecision in the data and other influences not captured by distance to road, the probability of human presence is only significantly related to distance to road for the Ndoki-Dzanga site for the separate site analyses.

Logistic regression [21] using the pooled elephant dung-count data from the Ndoki-Dzanga, Boumba Bek, Salonga, and Odzala-Koukoua surveys indicated a significant positive relationship between the probability of presence of elephants and increasing distance from the nearest major road (Figure 2A). The data for Minkébé were omitted from this analysis because, unique to this site, forest elephant dung was recorded on all transects regardless of the distance from a road, and therefore the data were not informative for logistic regression. Model results were improved by including site as a factor covariate. The exceptions were Ndoki-Dzanga and Odzala that not only had the same slope, but also the same intercept term. Odzala-Koukoua and Ndoki-Dzanga consistently had the highest probability of elephant occurrence at all distances from the nearest road, with intermediate probability for Boumba Bek. Salonga, where elephant dung was recorded on just 22 out of 130 line transects, had the lowest probability of elephant occurrence (see Figure 2A). Performing separate logistic regression analyses on each site's data confirmed the relationship between the probability of elephant occurrence and the distance from the nearest road, except for the Salonga site (see Figure 3), in which distance from the nearest road had no effect on the probability of elephant dung occurrence.

Using the human-sign data pooled across the same MIKE survey sites, but this time including Minkébé, we found that the probability of human presence decreased with increasing distance from the nearest road, in contrast to the probability of elephant occurrence (Figure 2B). However, the probability of human presence was not as dissimilar between the five sites as was the probability of elephant occurrence. In this case, Ndoki-Dzanga and Odzala were the most dissimilar, having the highest and lowest probability of human presence at all distances from the nearest road, respectively. Minkébé, Salonga, and Boumba Bek occupied the middle ground in terms of the probability of human presence and were not significantly dissimilar from one another. Like human sign, the encounter rate of poached elephant carcasses decreased with distance from the nearest road (Spearman correlation coefficient *ρ* = −0.663, *n* = 13, *p* = 0.014), and no poached carcasses were found beyond 45 km of a road.

**Figure 4:**
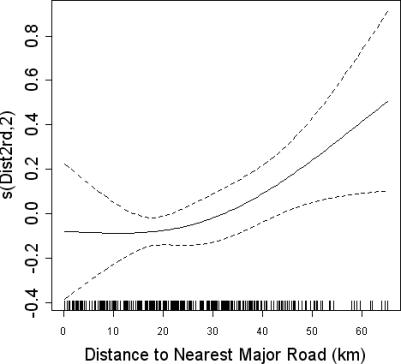
Estimated Conditional Dependence of Elephant Dung-Pile Numbers on Distance to Road (in Kilometres) Estimates (solid line) and confidence intervals (dashed lines), with a rug plot indicating observation density along the bottom of the plot, are shown. To avoid over-fitting, the degrees of freedom were restricted to two for the distance-to-road covariate.

Generalized Additive Models [22] provide a flexible, non-parametric technique for modelling the extreme variation in the elephant dung counts. Conditioning on elephant presence, the results indicate a significant positive relationship between elephant density and distance from roads. However, including the site covariate in addition dramatically increased the deviance explained from 22.5% to 95.4% and reduced the Generalized Cross Validation (GCV) score [23] (which is equivalent to Akaike's Information Criterion), from 14.734 to 6.742. Figure 4 illustrates the estimated conditional dependence of elephant dung-pile numbers on distance from road. The significant difference between the MIKE sites highlighted by the site covariate indicates that there are site-specific ecological influences or additional local human pressures not captured by distance to the nearest major road.

### Megatransect Data

**Figure 5:**
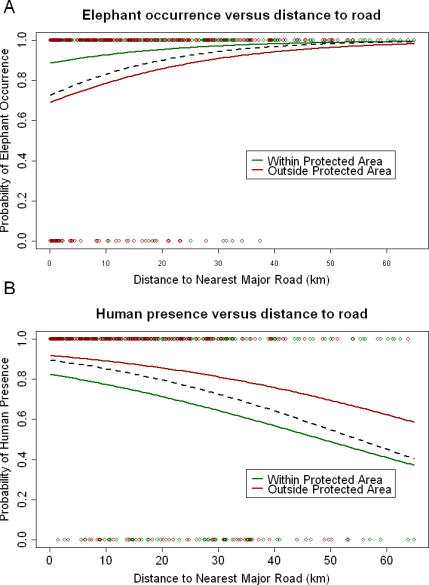
Results of Fitting a Logistic Regression Model to Elephant and Human Presence/Absence Megatransect Data Distance to road (in kilometres) and location within or outside the protected areas were used as explanatory variables. (A) shows the elephant data, and (B) shows the human data. The observations and regression lines are colour-coded to correspond to within or outside the protected areas and the dashed line shows the regression line with only the distance to road covariate. The covariates distance to road and location within or outside the protected areas are significant for both elephant and human probability of occurrence.

The scale of the Megatransect transcended site-level surveys and thus provided a useful extensive comparison to the more intensive, but localised, MIKE surveys. The Megatransect also traversed six protected areas, which allowed the effect of protected area status on forest elephants and human presence to be examined. Applying logistic regression [21] to the Megatransect data indicated a significant relationship between the probability of presence of elephants and the distance from the nearest road (Figure 5A), consistent with the analysis of the MIKE dataset. Model results were not improved by including distance to the nearest protected area boundary as a covariate, but they were significantly improved by including a binary factor covariate describing whether or not the count data were collected within or outside of a protected area. Although the pattern of response of the probability of elephant occurrence to increasing distance from road is similar for within and outside of protected areas, protected areas consistently had the highest probability of elephant occurrence at all distances from the nearest road (Figure 5A).

Consistent with MIKE survey data, the probability of human presence on the Megatransect decreased significantly with increasing distance from the nearest road in contrast to the probability of elephant occurrence, and was consistently lower inside protected areas compared to outside for all distances from the nearest road (Figure 5B).

**Figure 6:**
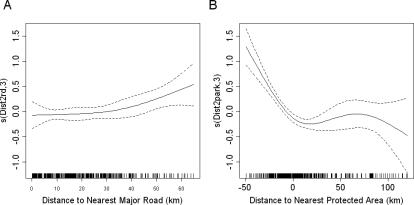
Estimated Conditional Dependence of Elephant Dung-Pile Numbers on Distance from Road (in Kilometres) and Distance to the Nearest Protected Area Boundary (in Kilometres) (A) shows the effect of distance from the road, and (B) shows the effect of distance to the nearest boundary of the protected area. Negative distances indicate locations inside protected areas. Estimates (solid lines) and confidence intervals (dashed lines), with a rug plot indicating observation density along the bottom of the plot, are shown. To avoid over-fitting, the degrees of freedom for this model were restricted to 3 for both covariates.

Generalized Additive Models [22] were applied to the elephant dung counts from the Megatransect while conditioning on elephant presence. The results indicate a significant relationship between elephant dung counts and both distance from roads and distance to protected areas. However, in contrast to the model fit to the MIKE data, this model is only able to explain 19.7% of the deviance. Figure 6 illustrates the estimated conditional dependence of elephant dung-pile numbers on distance from road (Figure 6A) and distance to protected areas (Figure 6B) that shows a positive relationship with increasing distance from roads and a negative relationship for increasing distance from protected areas.

## Discussion

Our surveys confirmed the observations of conservationists [24] that numbers and range of forest elephant populations are in decline and that they continue to be poached for ivory, and probably meat, including inside NPs. In common with previous work in the Congo Basin [13], distance from the nearest road was a strong predictor of forest elephant abundance, human presence, and levels of poaching.

**Figure 7:**
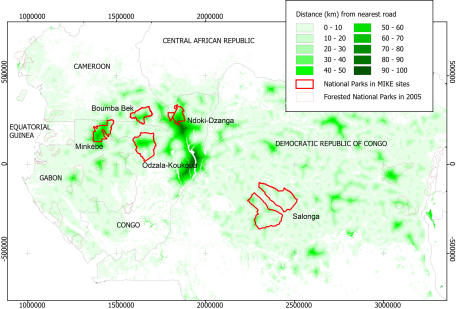
National Parks in MIKE Sites, the Forested National Parks of Central Africa, and Their Isolation from Roads

Within the consistent pattern of increasing elephant abundance and decreasing human-sign frequency with increasing distance from roads, site-level differences were variable and informative. Minkébé was the only site in which elephant dung was recorded on all transects. For other sites, the probability of occurrence decreased in the order Odzala-Koukoua, Ndoki-Dzanga, Boumba Bek, and finally Salonga. Elephant density by NP decreased in the same order, which is consistent with the remoteness of sites from the nearest road (Figure 7). Total NP area was not correlated with elephant density; however, there was a significant positive correlation between the area of parks that was over 40 km from a road and mean elephant density (*ρ* = 0.9, *n* = 5, *p* = 0.037). Thus, although Salonga NP is close to three times bigger than any other park surveyed, it comprises two separate sectors with some 46% of the total surface area within 10 km of a road, and nowhere in the park is beyond 40 km from a road. By contrast, just 0.7% of the Minkébé NP is within 10 km of a road, and a full 59% is more than 40 km from a road. Only in Minkébé and Odzala-Koukoua NPs do areas exist that are more than 60 km from the nearest road.

It is noteworthy that the road system of Salonga NP, which was well developed during colonial and immediately post-colonial times, has gradually fallen into disrepair, and today, the roads are used primarily as footpaths. In all other MIKE sites surveyed, the closest roads to the site are open to regular vehicular traffic, and many have been opened within only the last 10–20 y. Salonga has, therefore, a longer history of penetration by roads than other sites, which may be reflected, not only in the dearth of elephants, but the distribution of human signs, which were more likely to occur further from roads rather than closer to them. The long-term accessibility to the forest and heavy hunting in Salonga, including hunting for elephants [10], appears to have extirpated wildlife close to roads, forcing hunters to become more active in the most-remote areas of the park. Several navigable rivers also run through Salonga NP, which provide access and may confound an effect of roads as a proxy for isolation.

**Figure 8:**
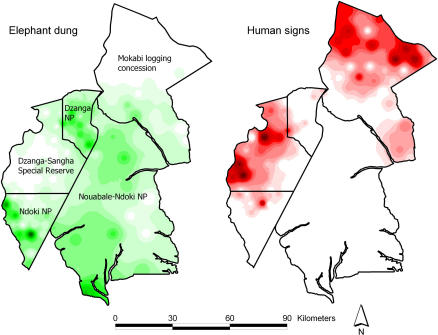
Interpolated Elephant Dung Count and Human-Sign Frequency across the Ndoki-Dzanga MIKE Site Increasing colour intensity signifies increasing dung and human-sign frequency.

The trends observed in the other MIKE sites (Figure 3) indicate that they have not yet reached such an advanced state of degradation as Salonga because strong relationships still exist between elephant abundance, human-sign frequency, and distance from the nearest road. Elephants still occur in moderate to high densities in remote areas, and at an exceptional density in Minkébé. However, it is clear that elephants are being concentrated into the most-remote sectors of all sites in a near-perfect juxtaposition with the distribution of human activity as exemplified by the simple interpolations of human-sign and elephant dung frequency from Ndoki-Dzanga (Figure 8). This startling image is reminiscent of Parker and Graham's description of savannah elephant distribution as the “negative” of human density [25], which was identified as a major factor in the decline of the elephant in Eastern Africa. Without effective management intervention to reduce fragmentation of remote forests [26], the human–elephant interface will move deeper into the forest, and elephants will continue to retreat into an increasingly less-remote core in the face of an advancing “human front.”

It is important to remember that the MIKE sites likely represent the “best-case” conservation status scenario because they were deliberately chosen from among the longest-established protected areas in some of the most-remote locations in Central Africa. Landscape-level conservation plans, which include conservation measures to reduce hunting and trafficking of bushmeat along roads, have been underway in Minkébé, Ndoki-Dzanga, Odzala-Koukoua, and Boumba Bek for at least a decade, and even Salonga has benefited from some conservation effort. Most of the remainder of the Congo Basin does not receive any tangible wildlife management, and the conservation status of forest elephants is probably considerably worse. A simple analysis of the degree of fragmentation caused by roads across the range of the forest elephant is revealing (Figure 7). In the 1,893,000 km^2^ of potentially available forest elephant habitat in the Congo Basin, some 1,229,173 km^2^ (64.9%) is within 10 km of a road. Just 21,845 km^2^ is over 50 km from the nearest road in just three countries, Congo, Gabon, and the Democratic Republic of Congo. Only Congo has potential elephant habitat beyond 70 km from a road, in the vast Likouala swamps to the northeast of the country. The road shapefile (Environmental Systems Research Institute [ESRI]) used is also restricted to major roads and thoroughfares, since most logging roads are either not geo-referenced or not mapped. Therefore the true degree of fragmentation of Central Africa's forest is considerably worse than is depicted on this map.

Figure 7 indicates that the current NP system in the Congo Basin does a reasonable job of capturing the most remote tracts of forest that remain (with the exception of the Likouala swamps). Despite considerable budgetary increases in recent years, funding for NPs and conservation landscapes remains below that necessary for even minimal management [27,28], and an appropriate question to ask is whether or not protected areas actually protect forest elephants. The Megatransect data suggest strongly that NPs and protected areas are making a positive contribution to conservation because at any given distance from the nearest road, protected areas have (1) lower incidence of human sign, and (2) higher incidence of forest elephant sign than non-protected forest, at least in Congo and Gabon.

The situation in the rest of the protected areas system and the forest at large is likely to be considerably worse, particularly in areas of armed conflict, civil disorder, and deteriorating socio-economic conditions [29]. In the Ituri Forest of eastern Democratic Republic of Congo, for example, where some of the bloodiest fighting seen in recent decades has occurred, an estimated 17,000 kg of ivory was evacuated from a 25,000 km^2^ forest block in a 6 mo period during 2003 [30]. Given a mean estimated weight of ivory from African elephants of 6.8 kg [31], this could represent some 2,500 elephants. There is no doubt that forest elephants are under threat from illegal killing across Central Africa's forests, and soon, the only elephants left to poach will be those that remain in the interior of a few remote, well-funded, and well-managed NPs in politically stable countries.

In this paper, we have shown that even with a near-universal ban of the trade in ivory in place, forest elephant range and numbers are in serious decline. This is in contrast to much of the recent literature on “the African elephant” that indicates generally stable or increasing populations in Eastern and Southern Africa [32], and in some cases, dramatic population growth and a “return of the giants” [33]. The decline of the ecologically, socially, morphologically, and genetically distinct forest elephant, (perhaps a separate species [18] or, at the very least, an “evolutionary significant unit” [34] worthy of high conservation status) has profound implications for the diversity and resilience of the African elephant. Given their vulnerability compared to savannah elephants, the wellbeing of forest elephants must be given priority when making decisions about elephant management on the continental scale. Key issues that fall into this category include the future of the ivory trade [35] and resource allocation for international support programmes, such as MIKE.

Forest elephants will continue to decline unless four immediate actions are successfully implemented. First, a national- and regional-scale approach to road development planning and construction is necessary in which reduction of fragmentation of Africa's last forest elephant strongholds is a central component. Second, law enforcement, including aggressive anti-poaching, of remaining priority elephant populations in NPs must gain the financial and political commitment required to ensure management success. Third, the illegal trade in ivory must be brought under control in elephant-range states, transit countries, and destination nations. Forth, effective partnerships must be developed with private logging and mining companies to reduce their negative impacts in the peripheries of protected areas and stop encroachment into NPs.

## Materials and Methods

### Survey methods.

Density estimates of forest elephants in MIKE survey sites were obtained from dung counts conducted on systematic line-transect distance sampling surveys [36] designed and analysed using the Distance 4.1 software package [37]. Distance sampling is a standard survey method for abundance estimation in both terrestrial and marine environments but, as far as we are aware, has never been used for ground-based surveys on foot on a scale approaching that of the present study, which comprised a total area of 60,895 km^2^ in some of the most remote and difficult terrain in forested Africa. Site boundaries were defined following discussions with the MIKE directorate, national wildlife directors, and site-based personnel, and were ultimately constrained by the total operating budget. Rivers, flooded forest, and swamps were excluded from site definitions because elephant dung piles cannot be surveyed in these habitats.

An attempt was made to design site boundaries that captured the gradient of human impacts on elephants, balanced against the need for a reasonable level of precision within each survey stratum. “Reasonable” precision was defined as a 25% coefficient of variation (CV) for estimates of elephant dung density for each survey stratum. To improve precision, each MIKE site was stratified according to expected elephant dung-pile encounter rate (*n*_0_/*L*_0_) based on either data from short pilot studies or from expert opinion in the case of the vast Salonga site, where a pilot study was prohibitively expensive. The effort in terms of total length of transect line required to attain the required precision was estimated according to the equation on page 242 of [36] using a value of three for the dispersion parameter *b* as recommended by Buckland et al. [36]:

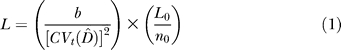
where *CV_t_*( D^) denotes the target CV for the density estimate.

Survey designs were completed using the “systematic segmented trackline sampling” option of Distance 4.1, as systematic designs with a random start are more robust to variations in the distribution of the population being sampled in terms of estimator precision [38]. This is a survey design class that superimposes a systematic set of parallel tracklines onto the survey region with a random start, along which line-transect segments are evenly spaced, again with a random start, at intervals and lengths determined by the user. Spacing and length of line transects varied by stratum and site according to the required sampling intensity. To potentially improve precision, line transects were oriented at 90° to major river drainages to run parallel to possible gradients in elephant density.

The start and end point of each line transect was uploaded to a Garmin 12XL GPS (global positioning system; http://www.garmin.com) to assist field navigation. If in the field, a line transect began in a swamp or river, it was displaced to the nearest location that could be found on terra firma. Similarly, when line transects traversed inundated areas, that portion of the transect was discarded, and an equivalent length was added to the end of the transect. Line transects were oriented using a sighting compass from the start point, and cut with a minimum of damage to the understorey. Observers walked slowly (ca. 0.5–0.75 km hr^−1^) along the line transect, scanning the ground for elephant dung piles. Distance along transects was measured using a hip-chain and topofil to the nearest metre, and the distance of the centre of each dung pile to the centreline were measured to the nearest centimetre using a 10-m tape measure. Survey methods are described in detail in [39].

In the field, the end of one line transect and the beginning of another were connected by reconnaissance walks following a “path of least resistance” through the forest [40]. On reconnaissance walks, a general heading was maintained in the desired direction of travel, but researchers were free to deviate to avoid thickets and steep hills or to follow elephant trails, human trails, and even logging roads. On reconnaissance walks, a continuous GPS tracklog is maintained, with a fix taken every 10–15 s. Data collection included all elephant dung piles, human sign, and vegetation type, and data records were coded by time (GMT). Data were later reconciled with GMT from the GPS tracklogs and thus geo-referenced and imported into ESRI ArcView 3.2 (Redlands, California, United States). Such reconnaissance walks are particularly useful for assessing the intensity and types of hunting activity if signs of humans are followed when encountered. However data are biased and provide only a general overview of large mammal distributions and human activity in the forest. The Megatransect also used reconnaissance survey methods consistent with the MIKE methods.

Elephant carcasses were defined as poached if evidence of hunting was obtained, which included gunshot holes in the carcass, removal of tusks, and meat on smoking racks. Elephant poaching camps were identified from other hunting camps by the presence of remains of elephant or very large meat-smoking racks.

### Analytical methods.

DISTANCE 4.1 software [37] was used to analyse the perpendicular distance data from the field measurements and to calculate the density of elephant dung piles by survey stratum and by individual line transect as described by Buckland et al. [36]. Different detection functions were fitted to the data sequentially using half-normal, uniform, and hazard rate key functions with cosine, hermite polynomial, and simple polynomial adjustment terms. The best model was selected on the basis of the lowest Akaike's Information Criterion score (AIC) [41], and *χ^2^* goodness-of-fit tests were used to examine the fit of the model to the data. On-site studies of elephant defecation and dung decay were not carried out due to the logistical and funding difficulties of working over such a large area, thus dung density was converted to elephant density using estimated conversion factors [42] of 19 defecations per day, and mean dung lifespan of 90 d for all sites.

In preparation for the statistical modelling, the centroid of each transect and 5-km Megatransect segment was used to calculate the distance of each “sample unit” from the nearest road or protected area boundary using the ESRI ArcView 3.2 extension “Nearest Feature” [43]. A shapefile of Central African roads was obtained from Global Forest Watch (World Resources Institute, Washington, D. C., United States). The protected areas shapefile was provided by the Wildlife Conservation Society.

Data from two MIKE sites, Dzanga-Sangha and Nouabalé-Ndoki, were pooled for analytical purposes since they are contiguous areas and therefore contained a single elephant population. Generalized Linear Models with a binary response and logistic transformation were used for the logistic regression analyses [21]. The Generalized Additive Models [22] fit to the dung-count data from the MIKE sites have the form

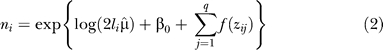
where *n_i_* denotes the number of dung piles detected on the *i* ^th^ transect, *l_i_* the length of the *i* ^th^ transect, and μ̂ is a site-specific estimate of the effective strip half-width [36] calculated using the Distance 4.1 software [37]. The term 2*l_i_* μ̂ gives the area effectively surveyed on transect *i*. *β*_0_ is the intercept, and *f*(*z_ij_*) is a smooth function of the *j* ^th^ covariate *z* associated with the *i* ^th^ transect. To deal with the over-dispersion in the data, a quasi-Poisson distribution was assumed. By including area effectively surveyed as an offset term in the model, dung density is, in effect, being modelled. The results are equivalent for elephant density if we assume constant conversion factors of 19 defecations per day and a mean dung lifespan of 90 d for all sites. The models were fit in R [44] using the mgcv package [45]. To avoid over-fitting, the degrees of freedom were restricted to two in the final model. The elephant dung-count data used in the analysis were over-dispersed in part due to the large number of zero counts. Some of these problems were eliminated by conditioning on elephant presence and only using non-zero counts for the analysis. In addition, using a quasi-Poisson model instead of a Poisson allowed for the modelling of over-dispersion by not assuming that the dispersion parameter is fixed at 1. The standard diagnostic plots used in model selection and assessment of fit indicated that the model is consistently giving lower fitted values when these are compared to the response values. The extraordinarily high elephant dung counts for certain areas of Minkébé, and occasionally for Odzala and Ndoki-Dzanga, that are in stark contrast to the counts at other sites or transects within the same site contribute to this problem. The same methods were applied to the Megatransect data except that the offset term representing the area effectively surveyed term was omitted since this dataset does not permit the estimation of the effective strip half-width μ̂ . Also, to avoid over-fitting, the degrees of freedom were restricted to 3 for both covariate terms in the final model for the Megatransect data. Spatial Analyst from ESRI was used to construct the images in Figure 7A and 7B, and the interpolations of human sign and elephant dung counts for Ndoki-Dzanga shown in Figure 8 were produced using the “Calculate Density” feature of the same extension.

## Abbreviations

MIKE: Monitoring of the Illegal Killing of Elephants
NP: national park
